# Inactivation of Gram-Negative Bacteria by Low-Pressure RF Remote Plasma Excited in N_2_-O_2_ Mixture and SF_6_ Gases

**Published:** 2013-12

**Authors:** Ayman Al-Mariri, Saker Saloum, Omar Mrad, Ghayath Swied, Bashar Alkhaled

**Affiliations:** 1 Department of Molecular Biology and Biotechnology, Atomic Energy Commission of Syria, Damascus, Syria;; 2 Department of Physics, Atomic Energy Commission of Syria, Damascus, Syria;; 3 Department of Chemistry, Atomic Energy Commission of Syria, Damascus, Syria

**Keywords:** Bacteria, Inactivation, Low pressure, Plasma, Polymer

## Abstract

The role of low-pressure RF plasma in the inactivation of *Escherichia coli* O157, *Klebsiella pneumoniae*, *Proteus mirabilis*, and *Enterobacter* *sakazakii* using N_2_-O_2_ and SF_6_ gases was assessed. 1×10^9^ colony-forming units (CFUs) of each bacterial isolate were placed on three polymer foils. The effects of pressure, power, distance from the source, and exposure time to plasma gases were optimized. The best conditions to inactivate the four bacteria were a 91%N_2_-9%O_2_ mixture and a 30-minute exposure time. SF_6_ gas was more efficient for all the tested isolates in as much as the treatment time was reduced to only three minutes. Therefore, low-pressure plasma could be used to sterilize heat and/or moisture-sensitive medical instruments.

## Introduction

The objective of the low-pressure plasma process is to control the generation of ions, electrons, and free radicals on a surface in order to modify its property. This process is now deemed a new attractive method in the field of sterilizing medical instruments.^1^ A low-pressure, 13.56-MHz hollow cathode discharge is a very attractive device for the process and synthesis of remote plasma-aided materials.^2^^,^^3^

Infections acquired in hospitals claim the life of one patient every 6 minutes. *Escherichia, Klebsiella, Proteus*, and *Enterobacter* species are the most common bacterial isolates that cause nosocomial infections,^4^^,^^5^ the treatment of which is severely hampered by antibiotic resistance.^4^ To overcome this, a great deal of research has been carried out on the effect of stresses such as cold shock, UV irradiation,^6^ and ozone on various bacteria^7^ and spores^6^ and the results have shown that exposure to such stresses bring about changes in the cell structure of these microorganisms. O_2_-N_2_ plasma mixture is a good example of such applications in that it is an efficient source of both N and O atoms (chemically reactive species) and of UV radiation emitted by NO-excited molecules.^8^

We sought to study the inactivation potency of plasma treatment by using O_2_-N_2_ and SF_6_ gases against local *E. coli* O157, *K. pneumonia*, *P. mirabilis*, and *E.* *sakazakii* bacterial isolates.

## Materials and Methods

*Plasma System*

The experimental set-up of the HCD-L 300 system was described in detail in our previous works.^3^^,^^9^ Tables 1 and 2 summarize the plasma operation conditions using N_2_-O_2_ mixture and pure SF_6_ gas, respectively.

**Table 1:** Experimental plasma conditions for the inactivation processes of 109 CFU/ml of different types of bacteria using N_2_-O_2_ plasma mixture

**Exp**	**x (%) in** **N**_2_ **-x% O**_2_	**Gas flow N**_2_ **/O**_2_ **(sccm)**	**Pressure** **(mbar)**	**Power (W)**	**Treatment time** **(min)**	**Z** **(cm)**	**Substrate**
1	9	500/50	0.35	300	30	4.5	96-well plate
2	9	500/50	0.65	300	30	4.5	96-well plate
3	9	500/50	0.95	300	30	4.5	96-well plate
4	9	500/50	1.17	300	30	4.5	96-well plate
5	9	500/50	1.24	300	30	4.5	96-well plate
6	9	500/50	1.48	300	30	4.5	96-well plate
7	5	500/26	1.25	300	30	4.5	96-well plate
8	2	500/10	1.25	300	30	4.5	96-well plate
9	9	500/50	1.25	300	5	4.5	96-well plate
10	9	500/50	1.25	300	10	4.5	96-well plate
11	9	500/50	1.25	300	15	4.5	96-well plate
12	9	500/50	1.25	300	20	4.5	96-well plate
13	9	500/50	1.25	300	25	4.5	96-well plate
14	9	500/50	1.25	300	40	4.5	96-well plate
15	9	500/50	1.25	300	30	4.5	PVC, PE, PET

**Table 2:** Experimental plasma conditions for the inactivation processes of different types of bacteria using pure SF_6_ plasma

	**Gas flow** **(sccm)**	**Pressure** **(mbar)**	**Power** **(W)**	**Treatment time** **(min)**	**Z ** **(cm)**	**Substrate**
1	200	0.55	100	0.5	4.5	96-well plate
2	200	0.55	100	1	4.5	96-well plate
3	200	0.55	100	3	4.5	96-well plate
4	200	0.55	100	5	4.5	96-well plate
5	200	0.55	100	10	4.5	96-well plate
6	200	0.55	100	15	4.5	96-well plate

*Polymers*

Polyethylene (PE), polyethylene terephthalate (PET), and polyvinyl chloride (PVC) polymers, commercially used for bio-application, were provided as films. 

*Micro-Organisms and Growth Conditions*

Clinical local isolates were collected from patients suffering from urinary tract infection (*E. coli* O157 or *P.* *mirabilis*), upper respiratory tract infection (*K. pneumonia*), or gastrointestinal infection (*E.* *sakazakii*). Identification of the bacteria was performed by using the API20E method (bioMérieux, Charbonnieres-les-Bains, France). The isolates were grown using standard cultures (Difco, BD, Spars, MD), and the cultures were harvested in a sterile PBS and adjusted by spectrophotometry to 1.0×10^10^ CFU/ml. Serial dilutions of 100 µl (1.0×10^9^ CFU/ml) of each freshly grown isolate were placed either in 96-well microtiter plates or on three sterilized polymer foils. The plates and the foils were exposed to different experimental plasma conditions (tables 1 and 2). After treatment, the bacterial suspensions were grown on bacterial mediums. The plates were incubated for 24 hours at 37^°^C. All the experiments were confirmed in duplicate. Reported values were the average of each two values.

*Statistical Methods*

The statistical analyses were performed with SPSS statistical program (version 15). A mean value for each bacterial count was obtained by averaging the duplicate values after log conversion.

## Results

**Figure 1:**
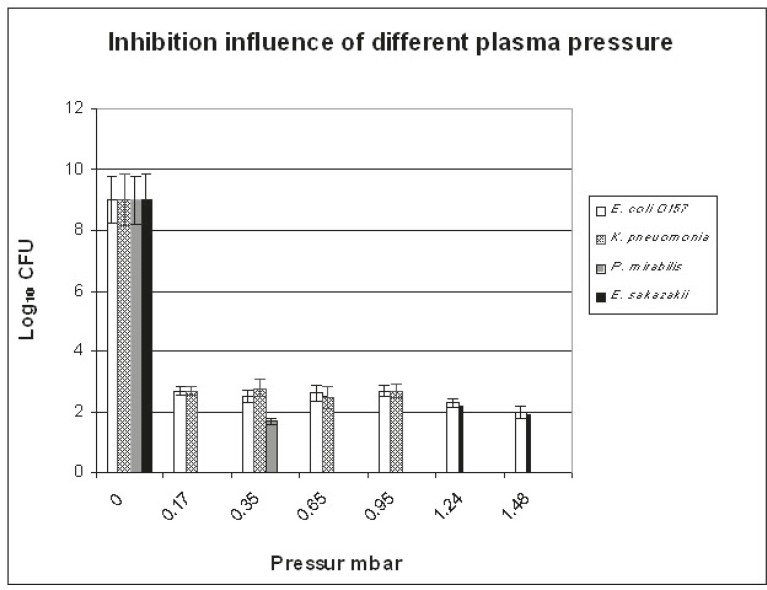
This is a depiction of the influence of pressure change using O2-N2 plasma mixture for 30 minutes against E. coli O157, K. pneumonia, P. mirabilis, and E. sakazakii on the standard medium

**Figure 2:**
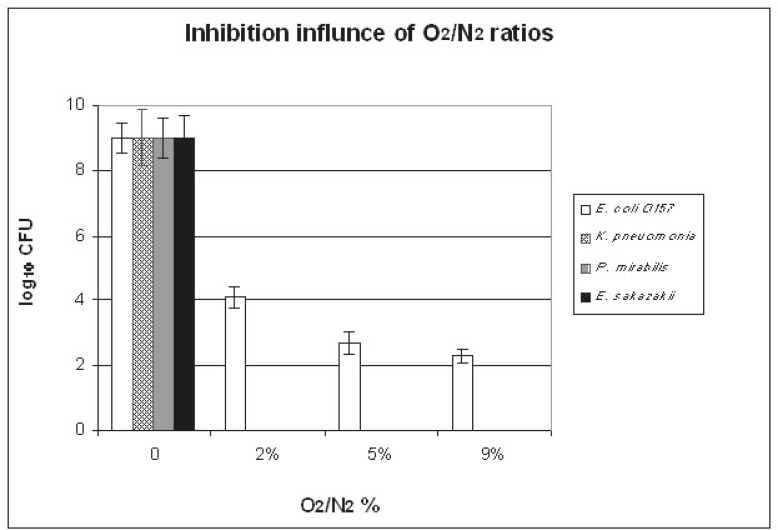
This is an illustration of the influence of oxygen percentage using O2-N2 plasma mixture for 30 minutes against E. coli O157, K. pneumonia, P. mirabilis, and E. sakazakii on the standard medium

**Figure 3:**
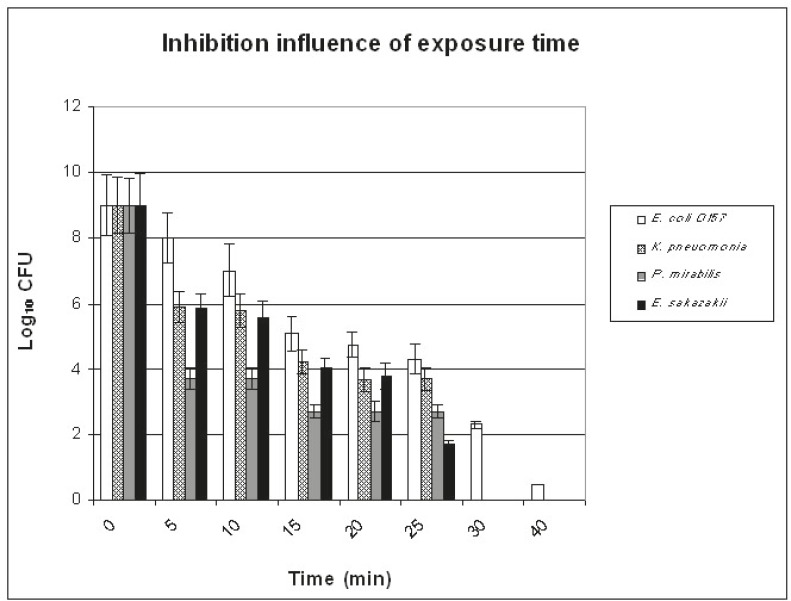
This is a depiction of the influence of the time of treatment using O2-N2 plasma mixture at 1.24 mbar pressure against E. coli O157, K. pneumonia, P. mirabilis, and E. sakazakii on the standard medium

The best conditions that led to the elimination of 10^9^ CFU/ml of each tested bacterial isolate (using O_2_-N_2_ plasma mixture at 300 W) are shown in figures 1, 2, and 3. Figure 1 illustrates the influence of plasma pressure on bacterial count (exp. 1-6 in table 1). Minimum CFU values were seen using 1.24 mbar pressure. The effect of O_2_ percentage (exp. 5,7, and 8 in table 1) in N_2_-x%O_2_ plasma mixture is presented in Figure 2: the CFU values of *E. coli* O157 were decreased, while O_2_ percentage was increased and only 2% O_2_ pressure was sufficient to completely deactivate the other types of bacteria. The influence of the time of treatment (exp. 5 and 9-14 in table 1) is demonstrated in figure 3. A 30-minute treatment was required to eliminate all the different kinds of microorganisms except* E. coli* O157, which was decreased only to 2×10^2^ CFU/ml. According to these results, the best conditions were 4.5 cm distance from the source, 30 minutes of treatment, 9% of O_2_, and 1.25 mbar pressure.

**Figure 4:**
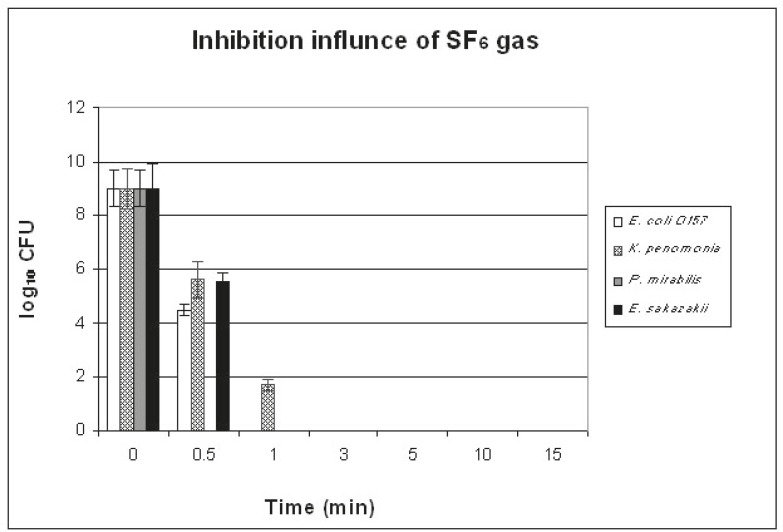
This is an illustration of the influence of the treatment with SF6 for one minute against E. coli O157, K. pneumonia, P. mirabilis, and E. sakazakii on the standard medium

Using the above-mentioned conditions on PVC, PE, and PET polymers (exp. 15 in table 1), we observed total inactivation of all the tested microorganisms with the PVC and PE polymers. However, *K. pneumonia* was not inactivated when we used PET polymer. 

Figure 4 shows the effect of SF_6_ plasma on all the previously mentioned microorganisms, using 96-well plates. Total inactivation of all the tested bacteria was seen only 3 minutes after the application of SF_6_. Approximately, 100% of all the *P.* *mirabilis* isolates were eliminated within 0.5 minute after SF_6_ exposure and 100% of all the *E. coli* O157 and *Enterobacter* isolates were eliminated within one minute after SF_6_ exposure. However, about 80% of the *K. pneumoniae* isolates were eliminated within one minute after exposure. 

## Discussion

Plasma treatment is considered a good and safe method to eliminate the decontamination of not only dental instruments but also general surgical instruments.^10^ Our results showed that the best bacterial inactivation plasma conditions were 300 W applied power, 4.5 cm distance from the source, and 1.24 mbar pressure at 9% of O_2_. Philip et al.^11^ demonstrated that total inactivation of *Bacillus subtilis* spores was achieved 40 minutes after plasma exposure at 100 W with 2% of O_2_. Furthermore, Xu et al.^1^ reported that the time needed for the inactivation of *Geobacillus stearothermophilus *spores was 3 minutes. In another study, Xu et al.^1^ also found that 10-20% of O_2_ was sufficient to inactivate these bacteria. Elsewhere, Feichtinger et al*.*^12^ discovered that spores numbers were reduced one second after the application of laboratory air as plasma gas. Our results agree with those reported by Xu et al.,^13^ who revealed that using argon (Ar) in a plasma jet source for 10 minutes did not totally eliminate* E. coli*. According to our results, O_2_-N_2_ gas using a plasma source was able to totally inactivate all kinds of bacteria except *E. coli*. The inactivation effect was more pronounced when we used flat polymers as substrates. Ricard and Monna^14^ reported that N_2_–5% O_2_ gas mixture completely eliminated *Streptococcus mutans*, *Porphyromonas gingivalis*, and *Prevotella intermedia* bacteria 15–20 minutes after treatment. In contrast, our results demonstrated that SF_6_ gas totally inactivated the bacteria in only 1-3 minutes. 

## Conclusion

Plasma inactivation using N_2_-O_2_ gas mixture and SF_6_ gas proved promising for the inactivation of the bacterial isolates in the present study. Our findings could be helpful in many medical and industrial fields; however, further investigations are needed to integrate this technique into the field of bacteria disinfection. 
